# Atypical *Salmonellosis* in a Horse: Implications for Hospital Safety

**DOI:** 10.1155/2020/7062408

**Published:** 2020-06-04

**Authors:** Kristina L. Rothers, Eileen S. Hackett, Gary L. Mason, Brad B. Nelson

**Affiliations:** ^1^Department of Clinical Sciences, Colorado State University, 300 West Drake Road, Fort Collins, CO 80523, USA; ^2^Veterinary Diagnostic Laboratory, Colorado State University, 300 West Lake Road, Fort Collins, CO 80523, USA

## Abstract

A 17-year-old Quarter Horse mare was evaluated for colic of 24-hour duration. Clinical signs and diagnostic evaluation were consistent with duodenitis-proximal jejunitis. The horse's clinical condition deteriorated despite medical treatment and was euthanized. Aerobic culture collected from small intestinal ingesta was positive for *Salmonella enterica* subsp. *enterica* serovar Hadar. *Salmonella* sp. is commonly implicated in nosocomial infections in equine veterinary hospitals usually through feces containing the organism. Considering *Salmonella* sp. was cultured from the jejunal luminal contents and the large volume of nasogastric reflux that was evacuated in this case, a perceived risk of *Salmonella* sp. transmission from infected gastric reflux to other hospitalized cases was realized. Infectious agent biosecurity precautions should be undertaken in horses with nasogastric reflux to prevent hospital-acquired transmission.

## 1. Introduction

*Salmonella* sp. infection in adult horses is typically associated with enterocolitis manifesting with diarrhea and hypoproteinemia [1]. Atypical presentations of salmonellosis can include signs such as voluminous gastric reflux and small intestinal ileus without diarrhea [1]. Horses can also be asymptomatic carriers, thereby shedding *Salmonella* spp. into the environment without any clinical signs of disease [2]. Nosocomial infections from *Salmonella* spp. are the most common source of disease transmission in equine hospitals [3, 4]. Horses presenting with fever, loose feces, and neutropenia or with presumptive diagnoses of anterior enteritis or colitis warrant the implementation of biosecurity protocols to minimize the risk of hospital-acquired infections [3–6]. *Salmonella* sp. is most commonly cultured from feces of horses with colic, and the potential risk of infection is usually attributed to fecal contamination disseminating throughout the facility [3, 4]. This identified risk has prompted routine surveillance fecal culturing as a method to detect shedding of the organism and enables the ability to segregate and maintain barriers between *Salmonella* sp. shedding horses and the general population [3, 7]. This report describes a horse with large quantities of gastrointestinal reflux, fever, and a diagnosis of enteritis attributed to an atypical *Salmonella enterica* serovar. The clinical and laboratory findings highlight the potential risk of disease transmission from sources other than feces and emphasize the importance of considering alternate sources of *Salmonella* sp. transmission.

## 2. Case Presentation

A 17-year-old Quarter Horse mare was referred to the Colorado State University Veterinary Teaching Hospital for colic of 24-hour duration. The horse was anorexic and lying down frequently the day prior to presentation. Findings from the referring veterinarian at that time were rectal temperature of 102°F (38.9°C), heart rate of 50 beats/minute (bpm), reduced borborygmi in all four quadrants, and 6 liters of net nasogastric reflux. Flunixin meglumine (1.1 mg/kg body weight (BW) IV) administration resulted in temporary improvement in clinical signs. Within 12 hours, the horse began showing mild signs of colic and was referred for further evaluation and treatment.

On hospital admission, the mare weighed 530 kg and had a rectal temperature of 102°F (38.9°C), heart rate of 54 bpm, reduced borborygmi in all four quadrants, and hyperemic mucous membranes. Following nasogastric intubation, 10 liters of net reflux was obtained. Transabdominal ultrasound examination revealed hypomotile, nondilated loops of the small intestine with 6-8 mm wall thickness and minimal peritoneal fluid in the ventral abdomen. Complete blood cell count examination identified leukopenia (4.6 × 10^9^/L; reference interval (RI): 5.5–10.5 × 10^9^/L) characterized by neutropenia (2.6 × 10^9^/L; RI: 3.0–7.0 × 10^9^/L) with a left shift (0.2 × 10^9^/L; RI: 0.0–0.1 × 10^9^/L) and slight toxic changes, as well as hyperfibrinogenemia (7.0 g/L; RI: 1.0–4.0 g/L). Serum biochemistry revealed hypophosphatemia (1.1 mmol/L; RI: 1.7–4.5 mmol/L), hypocalcaemia (1.09 mmol/L; RI: 1.15–1.40 mmol/L), hypomagnesemia (1.4 mmol/L; RI: 1.6–2.2 mmol/L), hyperproteinemia (80 g/L; RI: 58–74 g/L), hyperglobulinemia (5.1 mmol/L; RI: 2.5–4.5 mmol/L), and increased creatine kinase (6.6 mmol/L; RI: 1.0–4.7 mmol/L), aspartate aminotransferase (3.97 mmol/L; RI: 1.85–3.75 mmol/L) and gamma-glutamyl transferase (28 U/L; RI: 10–25 IU/L).

An intravenous jugular catheter was placed, and an initial bolus of 10 liters (20 mL/kg BW) of isotonic crystalloid fluids (Veterinary Plasma-Lyte A, Abbott, North Chicago, Illinois, USA) was administered. Crystalloid fluids were then continued at a rate of 3 L/hr (approx. 5.7 mL/kg BW/hr), with calcium gluconate (5.6 g/L) and magnesium sulfate (400 mg/L) supplementation. Additional treatments consisted of lidocaine (50 *μ*g/kg BW/min), polymyxin B (1,000 IU/kg BW IV q 12 hr), ranitidine (1.1 mg/kg BW IV q 8 hr), and flunixin meglumine (0.7 mg/kg BW IV q 12 hr). Gastric decompression was performed q 2 hr and resulted in approximately 4 L net reflux per hr.

After 18 hrs of medical treatment, rectal temperature was 102.5°F (39.2°C), and there was decreased borborygmi, hyperemic mucous membranes, and persistent colic characterized by pacing and muscle fasciculations. A venous blood gas revealed mild acidosis (pH 7.3) and decreased ionized calcium (1.2 mmol/L; RI: 1.3–1.9 mmol/L). Repeat ultrasound examination revealed 5-6 cm dilated and hypomotile small intestinal loops with 3-6 mm wall thickness. Abdominocentesis fluid was collected and was grossly serosanguinous. The peritoneal fluid had a total protein of 38 g/L and a total nucleated cell count of 1470 cells/*μ*L, with differential cell proportions of 40% nondegenerate neutrophils, 40% large mononuclear cells, and 20% lymphocytes. Despite the continued medical therapies described previously, clinical signs did not improve over the next 30 hours of treatment. After discussions of case progression and minimal response to medical treatments, the owner elected euthanasia.

**Figure 1:**
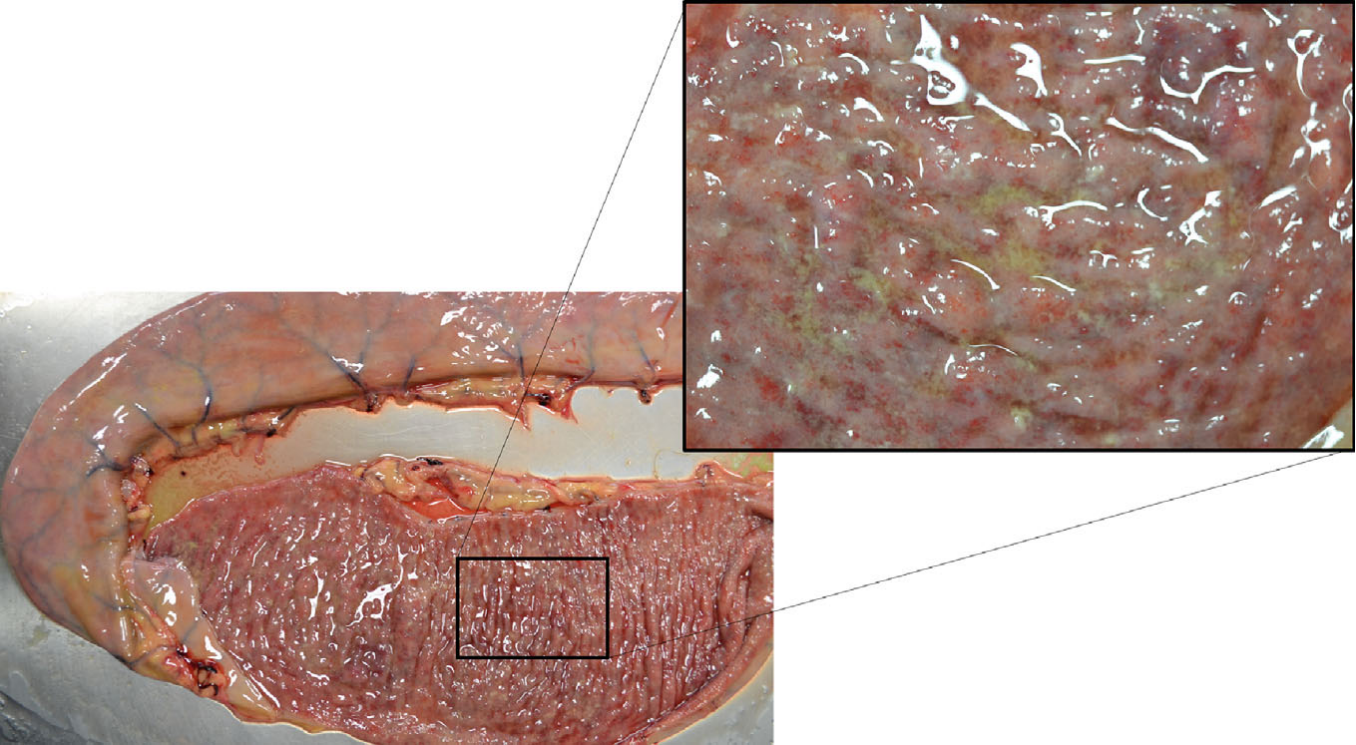
Gross appearance of the jejunal mucosa on cut section. Note the prominent edema, mural thickening, and reddening visible. The inset shows the jejunal mucosa magnified.

**Figure 2:**
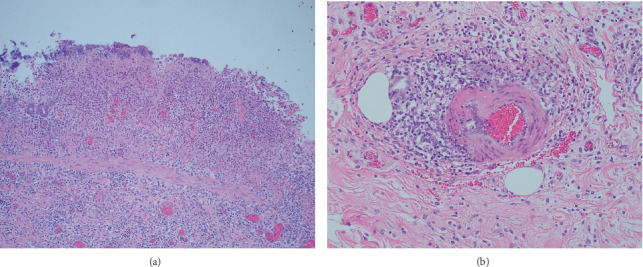
(a) Photomicrograph of the small intestinal mucosa and submucosa. The lamina propria and submucosa are expanded by chronic nonsuppurative inflammation with vascular congestion, acute erosive necrotizing enteritis, and bacterial colonization of necrotic surface debris. Hematoxylin and eosin, ×100 magnification. (b) Photomicrograph of the small intestinal submucosa. Periarterial nonsuppurative inflammation is visible, with acute changes including lymphatic dilation, edema, and segmental leukocytoclastic necrotizing arteritis. Hematoxylin and eosin, ×100 magnification.

Postmortem examination revealed diffuse edema and mural thickening of the jejunum and ileum (Figure 1). The mucosa had a dull reddish discoloration, and the mesenteric lymph nodes were hemorrhagic. There was also a mild red discoloration on the serosal surfaces of the duodenum to the cecum. The tissues were processed routinely for histopathology. Histopathologic findings revealed suppurative gastritis with ulceration, acute erosive necrotizing enteritis with Paneth cell metaplasia (Figure 2), colonic arteritis with thrombosis, and portal to centrilobular bacterial hepatitis. Jejunal luminal contents as well as fecal samples were submitted for aerobic culture. Samples were added to tetrathionate broth supplemented with iodine incubated at 42 degrees C overnight. Tetrathionate was subcultured to Xylose Lysine Tergitol 4 (XLT4) agar and incubated at 35 degrees C overnight. A representative colony was identified as *Salmonella* spp. Using triple sugar iron agar and agglutination in poly-O antisera, the isolate agglutinated in C2 antisera. Serotyping was performed by a reference laboratory (National Veterinary Services Laboratories, Ames, IA, USA), which identified *Salmonella enterica* subsp. *enterica*, group C2, serovar Hadar (*Salmonella* Hadar). Growth of *Clostridium perfringens* was also isolated from the small intestinal contents.

## 3. Discussion

This report describes a horse with fever, nasogastric reflux, and a suspected diagnosis of duodenitis-proximal jejunitis (DPJ) with a positive anaerobic culture of *Salmonella* Hadar from small intestinal ingesta. Gross pathological features consistent with salmonellosis included diffuse fibrinous and hemorrhagic inflammation of the jejunum and ileum. Histological assessment further characterized the intestinal lesions as acute erosive necrotizing enteritis with chronic inflammation in the lamina propria and submucosa in conjunction with vascular congestion and bacterial colonization of necrotic surface debris [8]. While many of the features observed in this case are consistent with DPJ, the apparent lack of involvement of the duodenum is uncharacteristic of the disease [1, 9, 10].

The horse in this case presented with signs typical of DPJ including fever, voluminous nasogastric reflux, and colic signs that resolved shortly following gastric decompression. Clinicopathologic variables commonly encountered in cases with DPJ were also observed, though these electrolyte abnormalities can be variable depending on the stage of the disease process [9]. The peritoneal total protein was increased without elevation in the total nucleated cell count, and elevated peritoneal total protein has been associated with increased mortality in horses with DPJ [11]. The cause of DPJ in horses remains elusive [9]. Bacterial agents including *Clostridium difficile*, *Clostridium perfringens*, and *Salmonella* spp., as well as parasitic infections and toxins including mycotoxins (fumonisin B1), have all been implicated as potential etiologies based on their isolation from affected cases [9]. Though *Clostridium difficile* and its toxins are commonly implicated in the pathogenesis of DPJ [1, 12, 13], data to confirm this hypothesis is lacking [9].

Subsequent inflammation of the duodenum and proximal jejunum is characteristic of DPJ [1, 9, 10]. The inflammation reduces intestinal absorption while increasing intestinal secretions into the lumen causing distension, thereby compromising intestinal peristalsis and culminating in ileus. Varying degrees of dehydration and endotoxemia are present and reflect the severity and chronicity of the stage of disease [14]. Diagnosis is usually confirmed surgically or at necropsy with hyperemia, edema, hemorrhage, and necrosis involving the affected intestinal segments [9]. The horse in this case had lesions in the jejunum and ileum, consisting of edema and mural thickening. Culture of the affected small intestinal contents revealed *Salmonella* Hadar and *Clostridium perfringens*, while the fecal culture was positive for *Salmonella* Hadar. Additionally, there was hemorrhage in the mesenteric lymph nodes as well as Paneth cell metaplasia consistent with a nonacute disease process [15].

Salmonellosis typically manifests in adult horses as enterocolitis with acute severe diarrhea and protein-losing enteropathy [1]. However, horses may also be latent subclinical carriers that shed during stress or present to the hospital as neonatal foals with bacteremia [1]. *Salmonella enterica* ssp. *enterica* accounts for approximately 60% of all *Salmonella* subspecies and approximately 99% of the clinical and subclinical infections in warm-blooded animals [16]. Considered an opportunistic pathogen, *Salmonella enterica* sp. is transmitted by fecal-oral routes and colonizes sections of the gastrointestinal tract, disrupting normal physiologic processes of absorption and secretion [1, 8]. Commonly affecting the colon in horses, the disruption of the colonic wall permits protein loss and the inability to reabsorb water, leading to diarrhea. While this horse did have a neutropenia with a left shift likely due to neutrophil migration into the affected intestinal tissues as is common with salmonellosis, there was no diarrhea or hypoproteinemia observed. A majority of equine salmonellosis cases are associated with *Salmonella enterica* serovars Typhimurium, Newport, Javiana, Braenderup, Anatum, Infantis, Muenchen, and Mbandaka [3, 6, 17, 18]. *Salmonella* Hadar is a rare serovar encountered in horses with intestinal disease and to our knowledge has not been detected in horses outside of the Netherlands [19, 20]. More commonly, *Salmonella* Hadar is associated with poultry and foodborne illness outbreaks in humans [21, 22]. Nonetheless, a clinical disease outbreak has occurred in a human maternity and neonatal hospital ward in the United Kingdom [23]. *Salmonella* sp. has zoonotic potential, and contaminated fluids and excretions from infected horses should be avoided, though it commonly does not impact humans outside of those with compromised immune systems.

*Salmonella* sp. outbreaks are a common source of nosocomial infections in equine hospitals [3, 4, 6]. Therefore, biosecurity protocols should be in place when an atypical presentation occurs. Monitoring equine hospital patient safety requires education, implementation of effective biosecurity protocols, and surveillance for contagious pathogens [3]. Using routine surveillance and raising awareness of cases that potentially shed *Salmonella* spp. are critical to prevent hospital-acquired infections. Once a case is identified, the patient can be isolated from the general population and additional precautions to prevent disease transmission are implemented. Identified risk factors of nosocomial colonization include exposure to hospitalized horses shedding *Salmonella* spp., either with direct contact or with environmental contamination, high ambient temperatures, treatment with antimicrobial agents, concurrent gastrointestinal tract disease, changes in diet, and use of contaminated equipment amongst patients (rectal thermometers, nasogastric tubes) [17]. An awareness of these risk factors will help guide individual biosecurity protocols to address these potential risks. In the report of *Salmonella* Hadar transmission in a human maternity ward [23], the index case was the mother of an admitted child with diarrhea and subsequent disease transmission was spread to 11 neonates over 3 months. Though predominantly neonates were infected, the duration of the outbreak suggested a difficulty in controlling disease propagation and a potential increased survivability of *Salmonella* Hadar in the environment despite routine hospital cleaning protocols [23].

Clinically normal horses or horses without diarrhea may have the organism in their feces and pose an infection risk to susceptible animals. Using barrier precautions, disinfecting common equipment, and being careful of disposal of feces and as this case suggests gastric reflux fluid can help reduce exposure to the general hospital population. The positive culture of *Salmonella* Hadar from the ingesta of the small intestine highlights the potential of recovered gastric reflux to be a source of *Salmonella* sp. transmission, especially when large volumes of gastric reflux are evacuated. In these patients without diarrhea, this gastric fluid could serve as a conduit for nosocomial infections. Thus, biosecurity protocols and procedures should not neglect the potential transmission routes of infectious disease in horses without diarrhea.

This case illustrates the importance of considering an infectious agent, namely, *Salmonella* sp., as an etiology for atypical clinical presentations of gastrointestinal disease. Despite the clinical signs being typical of DPJ, a diagnosis of enteritis with *Salmonella* Hadar infection was detected. The atypical presentation of *Salmonella* spp. detected in the jejunal fluid coupled with the large volume of reflux retrieved during gastric decompression highlights the importance of considering alternative nosocomial transmission pathways to protect the safety of hospital patients.

## Data Availability

There are no supplementary data for this article.
